# Editorial: Pathophysiologic Insights From Biomarker Studies in Neurological Disorders

**DOI:** 10.3389/fneur.2020.00151

**Published:** 2020-02-28

**Authors:** Stefan Bittner, Marcello Moccia, Tobias Warnecke, Tobias Ruck

**Affiliations:** ^1^Department of Neurology, Focus Program Translational Neuroscience (FTN) and Immunotherapy (FZI), Rhine Main Neuroscience Network, University Medical Center of the Johannes Gutenberg University Mainz, Mainz, Germany; ^2^Multiple Sclerosis Clinical Care and Research Centre, Department of Neuroscience, Reproductive Science and Odontostomatology, Federico II University, Naples, Italy; ^3^Department of Neurology with Institute of Translational Neurology, University of Münster, Münster, Germany

**Keywords:** biomarker, pathophysiology, neuroimmunology, neurodegeneration, pathomechanism

Neuroimmunological and neurodegenerative disorders are major healthcare issues, associated with high individual and socioeconomic burden. For many neurological disorders an early diagnosis is key for efficient management and therapy. However, neural tissues from brain, spinal cord, or peripheral nerves are difficult to sample, and their extraction is often associated with functional deficits. Therefore, in most cases a combination of biomarkers such as radiologic or biochemical findings are used to define a diagnosis in the clinical practice. The development of pathologically-sensitive and easy-to-measure disease biomarkers is a key factor for investigating new medications in phase II trials aiming to quickly screen their efficacy, and in phase III trials where they are coupled to clinical measures. Moreover, biomarkers are used to predict treatment efficacy and adverse event risk, paving the way for personalized medicine with individual risk assessment and prevention strategies.

In the last few decades the enormous technological progress, especially omics technologies, expanded the definition of biomarkers from biochemical and clinical, to genetic, proteomic, metabolic, or microbial markers. Interestingly, some of the biomarker studies additionally provided important insights into the pathophysiology of neurological disorders. In multiple sclerosis TNF-blocking drugs can promote onset or exacerbation of MS and GWAS (genome-wide association studies) data informed about the underlying mechanisms instructing clinical practice (1). In Parkinson's disease (PD), evaluation of Lewy pathology provided new pathophysiological insights attributing PD pathology progression to a prion-like process starting in the gastrointestinal tract (2). However, many other suggested biomarkers remain solely correlative, often lacking a causative link to the underlying disease mechanisms possibly explaining their lack in sensitivity and specificity.

This Research Topic reviews current knowledge and provides new insights into pathomechanisms of a wide variety of neuroimmunological and neurodegenerative disorders from studies on (novel) risk factors and outcome predicting biomarkers.

## Inflammatory Disorders of the Central Nervous System

There is still an unmet need for novel, innovative biomarker approaches in different stages of central nervous system inflammation ranging from first diagnosis to evaluation of inflammatory and neurodegenerative processes and therapy responses. In this Research Topic, Pawlitzki et al. shows the value of CSF-based biomarker approaches indicating ongoing chronic CNS inflammation even before first clinical sign of symptoms in patients with radiologically isolated syndrome, while Schwenkenbecher et al. demonstrate the importance of oligoclonal bands in the diagnostic algorithm of multiple sclerosis assessing the impact of the newest McDonald criteria. Until now, we have only a limited understanding of inflammatory and neurodegenerative processes within the CNS compartment in multiple sclerosis. Here, Birkner et al. assessed the role of ICAM-5, a CNS-restricted adhesion molecule, in mediating T cell-induced neuronal damage in the animal model of experimental autoimmune encephalomyelitis (EAE), Huhn et al. investigated the use of sodium MRI as a translational approach in MS patients. Another part of this Research Topic section focuses on the value of biomarker approaches in different real-world therapeutic settings, namely blood-based biomarkers in patients treated with fingolimod (Kaufmann et al.), dimethylfumarate (Manni et al.), and in a longitudinal setting in patients treated with natalizumab (Kaufmann et al.). The study by Vohl et al. investigates a cohort of patients treated with intrathecal corticosteroid therapy, a long-established, but sometimes under-appreciated therapeutic approach, in numerous centers specialized on the treatment of MS patients, that so far has suffered from the lack of large studies. In a cohort of 38 patients with autoimmune encephalitis (AE), Körtvelyessy et al. showed that CSF Progranulin levels were elevated during the acute phase, suggesting ongoing neuro-axonal damage, and that total-Tau (t-tau) and neurofilament light chain (NfL) levels were associated with the risk of clinical, laboratory, and radiological progression. Future studies on AE could benefit from the work of Amedonu et al., with the use of a novel assay to study molecular mechanisms of autoantibody-induced receptor internalization and to screen new molecular-based therapies.

## Neurodegeneration and Stroke

Neurodegenerative diseases have been conventionally studied in relation to the genetic risk of clinical/cognitive dysfunction and neuronal damage. In line with this, Goltermann et al. showed that apolipoprotein E (APOE) ε4 homozygosity, a well-known risk factor for Alzheimer's disease (AD), was associated with worse visuospatial working memory and global brain structural alterations in 62 non-neurological subjects from the FOR2107 Marburg-Münster Affective Cohort Study.

More recently, an interplay between inflammation and neurodegeneration has been recognized. Looking at conditions that are conventionally unrelated to inflammation, Hu et al. demonstrated an inflammatory component in the normal aging and in AD, by showing an accentuated shift from T-helper (Th)-1 to non-Th1 inflammatory pathways in the cerebrospinal fluid (CSF) (similar to what happens in multiple sclerosis). Hotter et al. evaluated 91 stroke patients not suffering from stroke-associated infections (derived from the PREDICT and 1000 Plus studies), and found that IL-6 was associated with several MRI measures of stroke severity (e.g., acute and final lesion perfusion and size), suggesting that inflammation is related to vascular parenchymal damage. Also, neuroinflammation during the fetal period could be related to neurodevelopmental disorders (e.g., autism spectrum disorder, schizophrenia). Cao et al. studied the role of α7nAChR on fetal sheep astrocytes, a classic model of fetal neurobiology, and further unraveled the complex interactions between inflammation and neurodegeneration at neurodevelopmental stages.

However, our Research Topic did not aim to underestimate clinical examination, which remains of utmost importance in the neurology clinical practice. In particular, in their review, Johnen and Bertoux suggested that the early clinical diagnosis of the behavioral variant frontotemporal dementia (bvFTD) requires an in-depth assessment of clinical signs, behavioral and psychological symptoms as well as cognitive performance. Authors strongly encouraged the design of novel neuropsychological tests covering understudied aspects of dementia (e.g., social and emotional processing, praxis abilities, interoceptive processing), and further expanding the number of clinical and neuropsychological biomarkers for the neurology clinical practice (Johnen and Bertoux).

## Movement Disorders, Parkinson's Disease, and Dysphagia

Over the last decade increasing scientific efforts have been made to establish biomarkers for early diagnosis of Parkinson's disease (PD) and other movement disorders. These potentially helpful biomarkers include findings from neuroimaging techniques, blood and cerebrospinal fluid (CSF) immune markers, technology-based objective measures (TOMs) provided by wearable devices, results from skin biopsies, and several others. Doppler et al. were now able to show that dermal phospho-alpha-synuclein (p-syn) in PD patients with glucocerebrosidase gene (GBA1) mutations seems to offer a similar distribution and frequency as observed in PD patients without a known mutation. Therefore, skin biopsy may be suitable to study p-syn deposition in these patients or even to identify premotor patients with GBA1 mutations (Doppler et al.). However, a panel of serum immune markers (i.e., cytokines) as analyzed using principal component analysis (PCA) was not found to allow for reliably predicting clinical PD subtypes (Yilmaz et al.). Varghese et al. introduced a technology-based system called Smart Device System (SDS) that was tested over 2 years to train an Artificial Intelligence System; this system will be further implemented for multi-modal high-resolution acceleration measurement of patients with PD or essential tremor (ET). Furthermore, Warnecke et al. used flexible endoscopic evaluation of swallowing (FEES) to detect specific pharyngolaryngeal movement disorders, such as arytenoid tremor that may help to differentiate multiple system atrophy (MSA) from PD.

In addition, biomarkers for detection of specific symptoms related to PD and other movement disorders have been studied throughout the last years. In their narrative review Prell et al. provide an overview of recent developments of bio-fluid and imaging biomarkers for dementia, depression, and fatigue in PD. They conclude that more research needs to be undertaken to find reliable combinations of predictors of these neuropsychological symptoms in PD on an individual level, and that standardization and harmonization of protocols in particular in CSF handling and neuroimaging has to be further implemented (Prell et al.). Richter et al. assessed the utility of brainstem raphe (BR) alterations evaluated by transcranial sonography (TCS) as a potential biomarker for apathy and depression in PD patients. These BR alterations correlated with depression and apathy scales indicating that a serotonergic signal disturbance might exist for both symptoms in PD (Richter et al.). Beside these neuropsychological symptoms, significant attention has being paid to gastrointestinal dysfunction and particularly swallowing disorders. Clinical research has shown that dysphagia is an often overlooked, but complex syndrome in many patients with neurodegenerative and neuromuscular disorders mainly contributing to disease burden in the majority of cases. Schröder et al. were able to show that reduced saliva substance P (SP) concentrations may predict early pharyngeal swallowing dysfunction in PD patients and may help to identify PD patients with a higher risk of developing clinically relevant dysphagia. Labeit et al. reported the first case of isolated dysphagia due to Jo-1 antibodies associated myositis. They suggest that myositis-focused diagnostics including an autoantibody panel should be done in patients with unclear dysphagia that show typical sings for myositis in the instrumental dysphagia assessment (Labeit et al.).

In the future biomarkers should not only be available for early diagnosis, but also for indicating responses to specific therapeutic interventions that help to guide treatment decisions in PD and other movements disorders. Schlenstedt et al. presented pilot data indirectly supporting the hypothesis that exercises specifically designed to target freezing of gait (FOG) might be more beneficial than non-FOG-specific interventions in PD patients. However, future studies should include larger samples and high frequency interventions to investigate the role of training balance performance to reduce the severity of FOG and to analyze which subgroup of PD patients shows the largest effect size (Schlenstedt et al.). Finally, Hatz et al. were able to demonstrate that deep brain stimulation of the subthalamic nucleus (STN) used to treat motor complications in advanced PD does not alter verbal fluency performance in a systematic way at group level. Nevertheless, when STN stimulation produces an alteration of verbal fluency performance in an individual PD patient, it was inversely correlated with left temporal delta power as measured by quantitative EEG (Hatz et al.).

## Disorders of the Peripheral Nervous System

The peripheral nervous system (PNS) consists of nervous structures (cranial and spinal nerves, enteric nervous system) and their associated receptors and organs. The diagnosis of PNS disorders is a multi-step process and often complicated by unspecific findings. Instructive cellular and non-cellular biomarkers for diagnosis, prognosis and/or treatment management are largely missing. However, recent years including studies of this Research Topic support the high relevance of biomarkers for an adequate disease management. Correspondingly, Stuhlmüller et al. review the role of myositis-specific and -associated antibodies in idiopathic inflammatory myopathies (IIM). They highlight their value in the diagnostic process and prognosis (organ involvement, risk for malignancies), and elaborate on detection methods and associated clinical phenotypes (Stuhlmüller et al.). De Paepe et al. demonstrate an upregulation of osmolyte accumulators and identify associated inflammatory signaling pathways in skeletal muscle cells upon pro-inflammatory stimuli *in vitro* and in skeletal muscle biopsies of IIM patients. The authors characterize specific osmolyte accumulators as potential biomarkers for regenerating muscle fibers or autoreactive immune cells (De Paepe et al.).

However, biomarker research might not only unravel associations with clinical parameters but also reveal important insights into pathophysiological disease mechanisms. With an unbiased proteomic profiling Roos et al. identified key molecules involved in the pathogenesis of sporadic inclusion body myositis (sIBM), which were further characterized by qPCR, immunostaining, immunofluorescence *in situ* and *in silico* studies. By these means, they were able to demonstrate a key role of specific macrophage subtypes and molecules (CD74, CD163, and STAT1) involved in their activation and type I interferon and interferon gamma associated pathways in sIBM (Roos et al.). With a similar proteomic approach Kölbel et al. identified candidate protein biomarkers in skeletal muscle of laminin-211 deficient type 1 A (MDC1A) congenital muscular dystrophy (CMD), one of the most devastating CMDs characterized by severe muscle weakness, brain abnormalities, and delayed motor milestones. The expression of 86 proteins associated with fibrosis and altered synaptic transmission was specifically linked to MDC1A skeletal muscle. Moreover, the observed alterations in metabolic pathways and mitochondrial function might identify new therapeutic targets (Kölbel et al.).

Changes in protein function, folding or metabolism is a common feature of several degenerative disorders of the PNS. In Marinesco-Sjögren syndrome (MSS) characterized by a clinical triad of bilateral cataracts, ataxia, and myopathy, mutations in the SIL1 gene lead to protein misfolding. However, read-out systems to identify pathogenic variants of SIL1 mutations are missing. Therefore, Gatz et al. introduce an *in vitro* system identifying the pathogenic consequences of different mutations on a cellular level. In the future, these findings can instruct genetic testing of patients with suspected MSS.

Besides disorders primarily affecting structures of the PNS, systemic autoimmune diseases such as Sjögren's syndrome might involve the PNS as well. In this Research Topic, Seeliger et al. provide a cross-sectional study of 144 patients with polyneuropathy of whom 44 were diagnosed with Sjögren's syndrome. They describe clinical findings and discuss potential tools to identify Neuro-Sjögren (Seeliger et al.). Other systemic inflammatory disorders such as vasculitides can also affect structures of the nervous system including the PNS resulting in a variety of potential symptoms. Strunk et al. review potential biomarkers in the context of nervous system vasculitides with a focus on body fluid derived markers and discuss their diagnostic and prognostic value.

Overall, the current Research Topic provides a deeper understanding of the pathomechanisms underlying neurological disorders and identifies new biomarkers and their pathophysiologic background. Thereby, the Research Topic might initiate hypothesis-driven refinement of current and novel biomarkers.

## Author Contributions

SB, MM, TW, and TR edited the Research Topic and drafted and revised the editorial manuscript. All authors read and approved the final version of the manuscript.

### Conflict of Interest

The authors declare that the research was conducted in the absence of any commercial or financial relationships that could be construed as a potential conflict of interest.
